# A DEPRESCRIBING CURRICULAR FRAMEWORK USING AN INTER-PROFESSIONAL APPROACH: IMPLICATIONS FOR NURSING EDUCATION

**DOI:** 10.1093/geroni/igad104.3773

**Published:** 2023-12-21

**Authors:** Winnie Sun, Cheryl Sadowski, Lalitha Raman-Wilms, Camille Gagnon

**Affiliations:** Ontario Tech University, Lindsay, Ontario, Canada; University of Alberta, Edmonton, Alberta, Canada; University of Manitoba, Winnipeg, Manitoba, Canada; Institut Universitaire de Gériatrie de Montréal, Montreal, Quebec, Canada

## Abstract

Background Deprescribing is an important component of managing polypharmacy and reducing harm from potentially inappropriate medications. Current undergraduate nursing education does not consistently incorporate components of deprescribing into curricula. It is essential to bridge the gap in promoting deprescribing competencies, teach related knowledge and skills and assess learning outcomes. The purpose of this presentation is to engage nursing educators who teach geriatrics to identify and implement a curriculum framework for deprescribing. Methodology The Canadian Medication Appropriateness and Deprescribing Network (CaDeN) Healthcare Professional Committee undertook a consensus approach to developing competencies for deprescribing, along with literature review and analysis of prescribing competencies. The authors outlined the required knowledge and skills related to the competencies, with recommended teaching and assessment strategies. Results The seven deprescribing competencies include: gathering and interpreting patients’ medication history and clinical information within their context, using tools that help identify potentially inappropriate medications, weighing potential benefit and harm of continuing or deprescribing medications, using shared decision-making about deprescribing, communicating deprescribing and monitoring plans, and monitoring progress and outcomes. Integrating deprescribing competencies in nursing curricula requires an intentional and structured approach across all years of the program, focusing on interprofessional collaboration. Learning activities should be active and practical, progressing from early to advanced learner skills and include integration of deprescribing through experiential education. Conclusion This presentation provides implications for the nursing profession to apply deprescribing competencies at different learner levels, using appropriate learning and assessment outcomes, and strategies in which deprescribing competencies could be achieved in an interprofessional setting.

